# Altered chromatin landscape and 3D interactions associated with primary constitutional *MLH1* epimutations

**DOI:** 10.1186/s13148-024-01770-3

**Published:** 2024-12-31

**Authors:** Paula Climent-Cantó, Marc Subirana-Granés, Mireia Ramos-Rodríguez, Estela Dámaso, Fátima Marín, Covadonga Vara, Beatriz Pérez-González, Helena Raurell, Elisabet Munté, José Luis Soto, Ángel Alonso, GiWon Shin, Hanlee Ji, Megan Hitchins, Gabriel Capellá, Lorenzo Pasquali, Marta Pineda

**Affiliations:** 1 Hereditary Cancer Group, ONCOBELL Program, Institut d’Investigació Biomèdica de Bellvitge (IDIBELL), L’Hospitalet de Llobregat, Spain; 2 Department of Medicine and Life Sciences, Universitat Pompeu Fabra, 08003 Barcelona, Spain; 3 Molecular Genetics Laboratory, Foundation for the Promotion of Health and Biomedical Research of Valencia Region (FISABIO), University Hospital of Elche, 03203 Elche, Alicante, Spain; 4 Genomics Medicine Unit, Navarrabiomed, Hospital Universitario de Navarra (HUN), Universidad Pública de Navarra (UPNA), IdiSNA, 31008 Pamplona, Spain; 5 Department of Medicine (Oncology), Stanford Cancer Institute, Stanford University, Stanford, CA 94305 USA; 6 Department of Biomedical Sciences, Cedars-Sinai Medical Center, Los Angeles, CA 90048 USA; 7 Ciber Oncología (CIBERONC), Instituto Salud Carlos III, Madrid, Spain; 8 Hereditary Cancer Program, Institut Català d’Oncologia (ICO), L’Hospitalet de Llobregat, Spain

**Keywords:** Lynch syndrome, Constitutional *MLH1* epimutation, *MLH1* promoter methylation, 3D interactions, Chromatin structure, *Cis*-regulatory regions

## Abstract

**Background:**

Lynch syndrome (LS), characterised by an increased risk for cancer, is mainly caused by germline pathogenic variants affecting a mismatch repair gene (*MLH1*, *MSH2*, *MSH6*, *PMS2*). Occasionally, LS may be caused by constitutional *MLH1* epimutation (CME) characterised by soma-wide methylation of one allele of the *MLH1* promoter. Most of these are “primary” epimutations, arising de novo without any apparent underlying *cis*-genetic cause, and are reversible between generations. We aimed to characterise genetic and gene regulatory changes associated with primary CME to elucidate possible underlying molecular mechanisms.

**Methods:**

Four carriers of a primary CME and three non-methylated relatives carrying the same genetic haplotype were included. Genetic alterations were sought using linked-read WGS in blood DNA. Transcriptome (RNA-seq), chromatin landscape (ATAC-seq, H3K27ac CUT&Tag) and 3D chromatin interactions (UMI-4C) were studied in lymphoblastoid cell lines. The *MLH1* promoter SNP (c.-93G > A, rs1800734) was used as a reporter in heterozygotes to assess allele-specific chromatin conformation states.

**Results:**

*MLH1* epimutant alleles presented a closed chromatin conformation and decreased levels of H3K27ac, as compared to the unmethylated allele. Moreover, the epimutant *MLH1* promoter exhibited differential 3D chromatin contacts, including lost and gained interactions with distal regulatory elements. Of note, rare genetic alterations potentially affecting transcription factor binding sites were found in the promoter-contacting region of CME carriers.

**Conclusions:**

Primary CMEs present allele-specific differential interaction patterns with neighbouring genes and regulatory elements. The role of the identified *cis*-regulatory regions in the molecular mechanism underlying the origin and maintenance of CME requires further investigation.

**Supplementary Information:**

The online version contains supplementary material available at 10.1186/s13148-024-01770-3.

## Introduction

Lynch syndrome (LS) is characterised by an increased risk of developing several types of cancers, mainly colon and endometrial tumours [1]. LS is mainly caused by germline genetic pathogenic variants in the mismatch repair (MMR) genes *MLH1*, *MSH2*, *MSH6,* and *PMS2* [2]. Rarely, constitutional epigenetic defects (constitutional epimutations) in *MLH1* and *MSH2* may be causative of LS [2].

Constitutional epimutations are associated with soma-wide monoallelic methylation of a promoter CpG island throughout normal tissues [3–7]. Constitutional epimutations are classified as secondary when linked to a genetic alteration in *cis*, or as primary when there is no apparent genetic cause [3]. Moreover, primary epimutations usually show de novo occurrence and/or erasure in the next generation [8, 9]. While only secondary constitutional epimutations have been reported for *MSH2* gene, all caused by germline deletions in the adjacent *EPCAM* gene [4], both primary and secondary epimutations of *MLH1* have been described.

Constitutional *MLH1* epimutations (CME) constitute a focal event that specifically and exclusively affects a region of 1.6 Kb (chr3:36,992,300–36,993,908) encompassing the CpG island spanning the bidirectional *MLH1* and *EPM2AIP1* promoter [10]. Secondary CMEs have been linked to rare point variants occurring within the promoter, exon 1 and intron 1 of *MLH1, Alu* insertions within the first *MLH1* exon, and structural variants affecting the entire *MLH1* gene, each resulting in varying levels of constitutional methylation [11–17]. However, most of the reported CME cases have not been associated with any *cis*-genetic variant located within the *MLH1* differentially methylated region (DMR) and have been classified as putative “primary” CME. Very rarely, CMEs have been associated with non-Mendelian transmission to offspring [8, 9].

Somatic *MLH1* promoter hypermethylation is one of the main causes of MMR deficiency in sporadic colorectal cancer (CRC) and endometrial tumours that show loss of MLH1 expression [18–20]. In *MLH1*-methylated CRC cell lines, Deng and colleagues defined a proximal region inside the *MLH1* promoter (referred to as C-D) in which methylation correlated with loss of *MLH1* transcription. Later studies showed that nucleosome occupancy at this region correlated with *MLH1* transcriptional silencing and preceded DNA methylation in RKO CRC cell lines [21].

Spreading of methylation from *Alu* elements within *MLH1* intron 1 towards the promoter has also been suggested as a potential underlying mechanism for *MLH1* methylation in CRC cell lines and tumours [22]. In CRC, hypermethylation of *MLH1* coexists with expression of the *BRAF* p.V600E variant [23], which is involved in the onset of the CpG island methylator phenotype (CIMP) through the action of the transcription factor (TF) MAFG [24–26]. On the other hand, the *MLH1* c.-93G > A promoter variant (rs1800734) has been associated with an increased risk of *MLH1*-methylated CRC [25, 27, 28] and *MLH1*-methylated endometrial cancer [29, 30]. It has been proposed that binding of TFAP4 to the wildtype c.-93G allele precludes the binding of MAFG and prevents the recruitment of the DNA methyltransferase DNMT3B to the *MLH1* promoter [24, 25].

The DMR in CME carriers is more confined compared to sporadic colorectal cancer [10]. While methylation is restricted to the shared *MLH1-EMP2AIP1* CpG island in carriers of a CME, multiple genes flanking *MLH1* in a 50 kb region are concomitantly hypermethylated in sporadic MSI, *MLH1*-hypermethylated CRC, leading to regional transcriptional silencing in these tumours [10, 31]. These differences in the observed methylation patterns suggest distinct mechanisms underlie the establishment of *MLH1* methylation in sporadic tumours and in CME carriers. In the present work, we delve into the mechanism underlying primary CME and their epigenetic consequences by analysing the chromatin landscape and 3D interactions of the epimutant and wildtype *MLH1* alleles along with genomic and transcriptome characterisation of de novo epimutation cases.

## Material and methods

### Patients and samples

Four CME carriers previously characterised by our group [10], showing hemiallelic methylation at the *MLH1* promoter in blood DNA samples, were selected for this study. Families 1 and 2 showed transmission of the genetic haplotype associated with methylation, but in an unmethylated state in the offspring, indicating intergenerational erasure of the epimutation (Supplementary Fig. 1). These cases were therefore classified as “primary” epimutations. In Family 4, the methylated haplotype was not transmitted, and in Family 3, haplotypes were not studied (Supplementary Fig. 1). Family 3 and 4 were consequently classified as putative primary epimutations (no evidence of methylation-associated haplotype inheritance) (Table 1 and Supplementary Fig. 1).

**Table 1:** Variants identified within the *MLH1* DMR and *MLH1* gene

Case ID (Dámaso et al., 2018)	Familial relationship	% Methylation in blood	*MLH1* promoter variants			Rare variants at DMR	Rare heterozygous variants in *MLH1*			
		(C-Deng region, MS-MLPA)	hg38 position	HGVS nomenclature	Allele in cis to the MAA in the CME carrier		hg38 position	HGVS nomenclature	SNP ID	Allele in cis to the MAA
ICO_1	Proband	56	chr3:36,993,455	c.-93G > A (het.)	c.-93A	–	–	–	–	–
–	Daughter of CME1	0	chr3:36,993,455	c.–93G > A (het.)	–	–	chr3:37,006,801	c.381-176dup	rs35239510	UP
CHN_1	Proband	47	chr3:36,993,455	c.-93G > A (het.)	c.-93A	–	chr3:37,026,448	**c.1409 + 441G > A**	rs182901684	c.1409 + 441A
							chr3:37,027,234	c.1409 + 1227C > T	rs1355851976	c.1409 + 1227C
–	Daughter of E2, sister of R.2.2	0	chr3:36,993,455	c.-93G > A (hom.)	–	–	chr3:37,001,778	c.306 + 725A > G	rs111823385	UP
							chr3:37,026,448	**c.1409 + 441G > A**	rs182901684	UP
–	Son of CME2, brother of R2.1	0	chr3:36,993,455	c.-93G > A (het.)	–	NA	NA	NA	NA	NA
HGUE_5	Proband	49	chr3:36,993,455;	c.-93G > A (het.);	c.-93A	c.-234_-236del (het.)	chr3:37,009,746	c.545 + 841A > G	rs562465919	c.545 + 841G
			chr3:36,993,312–36,993,314	c.-234_-236del (het.)						
HGUE_2	Proband	49	chr3:36,993,455	c.-93G > A (het.)	c.-93G	–	–	–	–	–

Results on constitutional *MLH1* epimutation (CME) carriers and relatives (R) are included. For each sample, the correspondence between case ID from Dámaso et al. (2018) and IDs used in this study is shown. Relationships between individuals are described in the familial relationship column. For each sample, levels of methylation in blood at the Deng-C region are indicated. Variants in the *MLH*1 promoter, as well as rare variants (AF < 1%) within the DMR and heterozygous rare variants in *MLH1* gene, are included. Variants were named according to HGVS guidelines and using MANE transcript NM_000249. Effect on splicing was calculated using SpliceAI. The deltaScore for each possible acceptor or donor event in the MANE transcript is shown. Rare variants in *MLH1* shared between CMEs and their relatives are indicated in bold. Sample R2.2 was not analysed by lrWGS. het. = heterozygous; hom. = homozygous; NA = not analysed; UP = unable to phase

All included CME carriers were heterozygous for the *MLH1* promoter variant c.-93G > A (population allele frequency 0.232, gnomAD v3.1.2) (Table 1), which was used herein as a reporter to distinguish epimutant from unmethylated alleles in sequence analyses. As previously reported [10], the methylation-associated allele (MAA) was linked to the c.-93A allele in 3 out of 4 CME carriers (CME1-3) and to the c.-93G allele in the remaining one (CME4); also, the CME3 patient carried a small deletion (c.-234_-236del) in *trans* to the MAA (Table 1). One adult child of CME1 (R1) and two adult children of CME2 (R2.1 and R2.2) who harboured the MAA in a non-methylated state were included as control relatives (Table 1 and Supplementary Fig. 1).

### Lymphoblastoid cell lines

Lymphoblastoid cell lines (LCLs) were used as the source material for the RNA-seq, ATAC-seq, CUT&Tag, and UMI-4C experiments. LCLs were established using Epstein Barr Virus as previously described [10]. Immortalised lymphoblastoid cells were grown at 37ºC in RPMI (Gibco #61870–010) supplemented with 10% FBS (Gibco #10270106), 1% Pen/Strep (Gibco #15140122) and 0.25 μg/mL Fungizone (Gibco #15290018).

### Linked read library preparation, sequencing, and analyses

Chromium 10X linked-read whole genome sequencing (10X Genomics, Pleasanton, CA, USA) [32] was performed using blood DNA samples from the four CME carriers and R1 and R2.1 control relatives. Sequencing libraries were prepared using the Chromium Library Kit (10 × Genomics, Pleasanton, CA, USA) following manufacturer’s protocol. Library was sequenced on an Illumina NovaSeq 6000 system with 150-by-150-bp paired-end reads. The resulting BCL files were demultiplexed and converted to FASTQ files using Long Ranger (v2.2.2) 'mkfastq'.

#### Alignment and SNVs/indels inference

Raw sequencing FASTQ output was aligned with BWA-MEM (v0.7.17) to the NCBI Human Reference Genome Build hg38 (hg38). Duplicates were marked using Samblaster (v0.1.24), and BAMs were sorted and indexed using Samtools (v1.9) [33]. Germline variants were called using: (1) GATK Haplotypecaller (v4.1.8.1), genotyped by GenotypeGVCFs and filtered using VariantRecalibrator and ApplyVQSR [34], and (2) Strelka2 (v2.9.10) [35] using the germline configuration. Only “PASS” variants identified by both callers’ algorithms were retained. The intersection between the two datasets was performed using BCFtools (v1.3.1) [36]. Variants were annotated using ANNOVAR (v20191024) (refGene, gnomad30 genome, avsnp150) [37, 38]. Only variants identified with a read depth greater than 5, a mapping quality exceeding 40, and an allele frequency higher than 30% (putative germline) were considered.

Further filtering was performed to screen variants mapping to the DMR (chr3:36,992,300–36,993,908), *MLH1* (chr3:36,993,226–37,050,896), *LRRFIP2* (chr3:37,052,626–37,183,689) and promoter-contacting region (chr3:36,596,059–37,430,058), which spans 396 kb upstream and 436 kb downstream of the DMR. Variants with a population frequency < 1% in gnomAD v3.0 genome database were considered as rare variants.

#### Phasing of SNVs and indels

To obtain phase information we used a well-established pipeline. Briefly, Long Ranger (v2.2.2) ‘wgs’ was run to align the reads to the hg38 reference genome. Variants called by GATK (v3.5) [39] using the-vcmode parameter were annotated with phasing information (phased block ID and phased genotype) in the resulting VCF files. All filters-passed variants near the *MLH1* gene (chr3:36,000,000–38,000,000) were collected and intersected with our previous final variant list.

#### Structural variants inference

Structural variants (SVs) were called using Delly (v1.1.6) [40], GRIDSS (v2.11.1–1) [41], Manta (v1.6.0) [42], and Smoove (v0.2.8) [43]. Only PASS variants were included, and ENCODE DAC blacklist was used to remove regions with anomalous, unstructured, and high signal/read counts. SVs were annotated as deletion, duplication, inversion, insertion, and breakends.

To control for population SVs, 2,504 low-coverage BAMs (hg38) and the PED file were downloaded from the 1,000 genomes AWS S3 bucket [44]. We extracted SV alignment evidence (discordant reads and split reads) from BAM control population using excord (v0.2.4) [45] with—discordant distance set to 500. Giggle (v0.6.3) and STIX (v1.0) were used to create an index and a database as described elsewhere [46]. The same methodology was applied to our patient cohort, and the inferred SVs were queried in both databases. SVs with evidence of > 9 counts in 1,000 genomes were defined as population variants and were removed. Coverage plots were generated for the remaining SVs using Samplot (v1.3.0) [47] and manually inspected.

#### Splicing prediction of identified variants

The potential effects of intronic variants on splicing were evaluated with the in silico tool SpliceAI [48] using max distance 2,000 bp for *MLH1* and 10,000 bp for *LRRFIP2* variants.

#### Insertion of transposable elements

MELT programme (v2.2.2) was employed to identify, annotate, and genotype non-reference Mobile Elements Insertions (MEIs), specifically *Alu*, LINE-1, HERVK and SINE-VNTR-*Alu* (SVA) elements. Default hg38 transposon files and parameters were used for the analysis. Only *Alu* insertions successfully passed all filters and were subsequently taken into consideration in the resulting VCF file. Heterozygous *Alu* insertions in CME carriers that were either absent or homozygous in control samples were considered as possible insertions and rearrangements.

#### Motif analysis

The package TFBSTools v1.36.0 [49] and JASPAR2022 (v.0.99.7) were used to predict binding sites for transcription factors with a minimum overlap score of 80% to the *MLH1* promoter sequence (chr3:36,992,079–36,994,100).

The package motifbreakR v.2.12.3 [50] was used to identify the SNVs and indels that disrupt TF binding based on position probability matrices (PPM). Default settings were applied, using a P value threshold of 1 × 10^–4^. We selected MotifDb (v.1.40.0) as the chosen TF motifs database [51]. Output motifs from FlyFactor Survey, ScerTF, stamlab, and versions older than HOCOMOCOv10 and JASPAR2022 were not considered. Only variants classified in the output file as “strong” based on the setup parameters were considered.

To identify the motifs potentially bound by transcriptional repressors and activators or insulator proteins, we considered TFs included in the Gene Ontology categories “DNA-binding transcription repressor activity”, “DNA-binding transcription activator activity”, and “chromatin insulator sequence binding” obtained from AmiGO [52, 53]. In this analysis, we did consider binding motifs of TFs with potential repressor activity for which the alternative allele score was higher than the reference allele, as well as TFs with activator activity for which the alternative allele score was smaller than the reference allele.

### RNA-seq library preparation and RNA isoform analysis

Total RNA from LCLs was isolated using Trizol (Ambion) and the RNeasy Mini Kit (Qiagen). RNA-seq libraries and 75-bp or 100-bp paired-end sequencing were undertaken at the Genomic Units from Centre Nacional d’Anàlisi Genòmica (CNAG) and Centre for Genomic Regulation (CRG) (Barcelona, Spain).

RNA-seq reads from FASTQ files were mapped to hg38 using Hisat2 (v2.1.0) [54], with the option --dta for downstream transcriptome assembly. Sorted and indexed BAM files were generated with samtools (v1.17) [33]. De-novo isoform discovery and quantification were performed using the StringTie (v2.2.1) [55] transcript assembler. First, StringTie was used to assemble the read alignments obtained in the previous step. A non-redundant set of transcripts from all RNA-seq samples was generated using the --merge option. Then, transcript abundances (TPMs) and read coverage tables were obtained for each of the input transcripts and annotated in a GTF file. SUPPA (v2.3) [56] was used to obtain the alternative splicing events from the GTF file and calculate the percent-splice-in (PSI) values using the transcript abundances per sample. The differential transcript usage between epimutant and control cells for *MLH1* and adjacent genes (*EPM2AIP2* and *LRRFIP2*) was calculated with SUPPA diffSPlice by applying a multiple testing correction test (FDR).

### ATAC-seq library preparation and data processing

ATAC-seq libraries were prepared as previously described [57]. For each experiment, 50,000 cells were collected and incubated in 300 µl cold lysis buffer (10 mM Tris–HCl pH 7.4, 10 mM NaCl, 3 mM MgCl_2_, 0.1% Igepal CA-630) for 25 min on ice. Nuclei were centrifuged for 15 min at 500 g at 4°C with low acceleration and brake settings. Then, the pellet was resuspended in 100 µL of the lysis buffer and centrifuged again. After centrifugation, nuclei were resuspended in 25 µL of reaction buffer containing 2 µL of Tn5 transposase, 12.5 µL of TD buffer (Nextera DNA Library Prep Kit, 15,028,212, Illumina), and 10.5 µL of water. Samples were incubated at 37ºC for 1 h. After incubation, reaction was inactivated by adding 5 µL of clean-up buffer (900 mM NaCl, 300 mM EDTA), 2 µL of 5% SDS, 2 µL of Proteinase K (ThermoScientific #EO0491) and incubated for 30 min at 40 °C. DNA was isolated using 2X SPRI beads clean-up (AgencourtAMPureXP, Beckman-Coulter, #A63880) and eluted in 21 µL 10 mM Tris–HCl pH8.

For library preparation, purified DNA was amplified performing two sequential PCRs with 9 cycles each. PCR mix was prepared as: 21 μL DNA, 25 μL NEBNext HiFi 2 × PCR Master mix, 2 µL of universal Ad1 primer and 2 µL of uniquely barcoded Ad2 primer [58]. Library was amplified in a thermocycler using the following programme: 72°C for 5 min, 98ºC for 30 s, 9 cycles of 98°C for 10 s and 63°C for 30 s, and a final extension at 72°C for 1 min and at 4°C hold. First amplification was purified for small fragment selection using 0.6X SPRI beads clean-up, following the manufacturer’s instructions. DNA was eluted in 21 µL 10 mM Tris–HCl pH8. The purified PCR product was amplified again using the same conditions and purified using 1.8X SPRI beads clean-up. Library sequencing was undertaken at the Genomic Units from CRG (Barcelona, Spain).

Reads were mapped to version hg38 of the human genome using Bowtie2 [59](v2.4.1) with default parameters. Next, duplicates and reads mapping to non-canonical chromosomes were removed using Samtools [33] (v1.10). Offset correction was performed using the ATACseqQC R package [60] (v1.22.0). Peak calling was performed using MACS2 [61] (v2.2.7.1) with arguments “--q 0.05 –nomodel --shift -100 --extsize 200”.

### CUT&Tag library preparation and data processing

CUT&Tag was performed as previously described [62] with minor modifications. Briefly, cells were harvested, counted, and centrifuged for 3 min at 600 g at room temperature. Cells were washed in Wash Buffer (20 mM HEPES pH 7.5, 150 mM NaCl, 0.5 mM Spermidine, 10 mM Sodium butyrate, 1X Protease inhibitor cocktail) and resuspended to 500,000 cells/mL. Concanavalin A coated (Bangs Laboratories, #BP531) magnetic beads were mixed with 10 volumes of binding buffer (20 mM HEPES pH 7.9, 10 mM KCl, 1 mM CaCl2, 1 mM MnCl2, 10 mM Sodium butyrate) and washed with 1.5 mL of binding buffer. Beads were resuspended in 1 volume of binding buffer and added to the cells. In each experiment, 100,000 cells and 10 μL of beads were used. The unbound supernatant was removed, and bead-bound cells were resuspended in 50 μL ice-cold Antibody buffer (20 mM HEPES pH 7.5, 150 mM NaCl, 0.5 mM Spermidine, 10 mM Sodium butyrate, 0.4 mM EDTA, 0.02% BSA, 0.05% Digitonin, 1X Protease inhibitor cocktail) and transferred to a LoBind tube. Primary antibody against H3K27ac (Abcam, #ab4729) was added (1:100) and incubated overnight on a rotating wheel at 4ºC. Next day, tubes were placed on the magnet stand to clear and the liquid drawn off. Secondary antibody (Antibodies Online, #ABIN101961) was diluted 1:100 in Dig-wash buffer (20 mM HEPES pH 7.5, 150 mM NaCl, 0.5 mM Spermidine, 10 mM Sodium butyrate, 0.05% Digitonin, 1X Protease inhibitor cocktail), added to the beads, and incubated on a rotating wheel at room temperature for 1 h. Tubes were placed on the magnet stand to clear and withdraw the liquid, and beads were washed three times with 1 mL of Dig-wash buffer. A 1:20 dilution of pA-Tn5 adapter complex (Cutana, 15–1017) was prepared in Dig-300 buffer (20 mM HEPES pH 7.5, 300 mM NaCl, 0.5 mM Spermidine, 10 mM Sodium butyrate, 0.01% Digitonin, 1X Protease inhibitor cocktail) and added to the beads. The tubes were mixed by gentle vortexing and incubated on a rotating wheel at room temperature for 1 h. After incubation, beads were washed three times in 1 mL of Dig-300 buffer. Tubes were placed on the magnet stand to draw off the liquid, and beads were resuspended in 300 μL of Tagmentation buffer (Dig-300 buffer, 10 mM MgCl_2_) and incubated at 37ºC for 1 h. To stop tagmentation and reverse cross-links, 10 µL 0.5 M EDTA, 3 µL 10% SDS and 2.5 µL 20 mg/mL Proteinase K were added to each sample and incubated for 1 h at 55 °C. DNA was purified by phenol–chloroform extraction and dissolved in 28 µL of TE (1 mM Tris–HCl pH 8, 0.1 mM EDTA).

For library amplification, 21 µL of DNA was mixed with 2 µL of a universal i5 and 2 μL of a unique barcoded i7 primer for each sample. A volume of 25 μLNEBNext HiFi 2X PCR Master mix was added to the mix. The following cycling conditions were used for library amplification: 72 °C for 5 min, 98 °C for 30 s, 13 cycles of 98 °C for 10 s and 63 °C for 10 s, and a final extension at 72 °C for 1 min and hold at 8 °C. Post-PCR clean-up was performed by adding 1.3X of Ampure XP beads (AgencourtAMPureXP, Beckman-Coulter, #A63880). The mix was incubated at ambient temperature for 5 min and washed twice gently with 80% ethanol. Samples were eluted in 25 µL 10 mM Tris–HCl pH 8. Library sequencing was undertaken at the Genomic Units from CRG (Barcelona, Spain).

Reads were mapped to hg38 using Bowtie2 [59] (v2.4.1) with parameters “–very-sensitive –no-mixed –no-discordant –phred33 -I 10—× 100”. Next, duplicates and reads mapping to non-canonical chromosomes were removed using Samtools [33] (v1.10). Peak calling was performed using MACS2 [61] (v2.2.7.1) with arguments “–broad –broadcutoff 0.1 –nomodel”.

### ATAC-seq, CUT&Tag and RNA-seq differential analysis

For the ATAC-seq and CUT&Tag data, the R package DiffBind [63] (v3.8.3) was used to load peaks called with -log_10_ *p*-value > 2 and create consensus peaksets, which were obtained by selecting regions present in more than 2 samples within the same condition (epimutation carrier or control). Then, condition-specific consensus peaks were merged.

RNA-seq reads were aligned to GENCODE version 38 using Salmon [64] (v1.3.0). Results were loaded into R using tximport [65] (v1.26.0), transcript information was summarised into genes, and protein-coding genes were retained for downstream analyses.

Differential analyses of ATAC-seq, CUT&Tag, and RNA-seq data were performed using de DESeq2 R package [66] (v1.38.1) with the design “ ~ group + condition”, where group refers to the family and condition to whether the sample is from an epimutation carrier or a control. Thresholds for defining statistically significant changes were set at adjusted *p* value < 0.1.

### UMI-4C

#### Library preparation

For each experiment, 4 million cells were collected and crosslinked with 1% paraformaldehyde in PBS for 10 min at room temperature and with gentle mixing. Glycine was added to a final concentration of 125 mM to quench the reaction. After 5 min, cells were washed twice with PBS and frozen. Cell pellet was incubated in 1 mL of cold lysis buffer (50 mM Tris–HCl pH 8, 150 mM NaCl, 5 mM EDTA, 1% Triton X-100, 0.5% NP-40, 1X protease inhibitor cocktail) for 30 min on ice with gentle mixing every 10 min. Following steps were performed as previously described [57] using the following upstream and downstream bait primers 5′-AGTGCCTTCAGCCAATCACC-3′ and 5′- TCAGTGCCTCGTGCTCA-3′. Libraries were sequenced on a NextSeq 500 using 2 × 75 bp reads.

#### UMI-4C processing and differential analysis

The UMI-4C reads were split based on the genotype of the reporter SNP c.-93G > A which was sequenced from the downstream bait primer. The allele-specific UMI-4C demultiplexed reads were then used as an input to UMI4Cats R package [67] to infer and quantify significant genomic interactions with the viewpoint. *MLH1* promoter interaction profiles were thus generated separately for the epimutant (methylated) and the wild-type (unmethylated) alleles using the contacts UMI4C() function. Next, contacts were mapped within a 1 Mb region (chr3:36,493,518–37,493,518) centred on the *MLH1* promoter using the makeUMI4C function with smoothing parameter min_win_factor = 0.03. Smoothed viewpoint-specific interaction profile plots were generated using the plotUMI4C function. Allele-specific differential contacts were inferred by Fisher’s exact test using the fisherUMI4C function with filter_low = 30 (FDR adjusted *p-*value 0.05).

## Results

### Absence of potentially causal genetic variants at the *MLH1* and *LRRFIP2* loci

We focus the study on four primary CME carriers (referred to as CME1 to CME4), for whom the presence of rare point variants in *cis* to the epimutation within the DMR spanning the *MLH1* CpG island had been previously ruled out by Sanger sequencing [10] (Table 1). Here, we extended the search for genetic variants across the entire *MLH1* gene by using linked-read whole genome sequencing (lrWGS). Three rare heterozygous intronic variants were identified within the *MLH1* gene in CME2 and CME3. Two of these variants (c.1409 + 441A in intron 12, c.545 + 841G in intron 6) are in phase with the methylation-associated allele (MAA) (Table 1). None of the identified variants were predicted to alter splicing (Supplementary Table 1). The variant in phase with the MAA in CME2 was shared with its relative R2.1 (Table 1), who inherited the same haplotype as CME2 but in an unmethylated state (Supplementary Fig. 1). Phasing of variants in R2.1 was not possible due to the lack of heterozygous variants within the *MLH1* promoter. Two other rare heterozygous variants were identified in the two control relatives but not in the CME carriers (Table 1 and Supplementary Table 1).

*MLH1* belongs to a trio of reverse-forward-reverse genes together with its neighbouring genes *EPM2AIP1* and *LRRFIP2*. Variants in *LRRFIP2* could potentially influence *MLH1* silencing through antisense transcription, as demonstrated for *PRDX1* variants and *MMACHC* methylation [68]. Sixteen rare intronic variants were detected in *LRRFIP2,* none of them predicted to have an impact on splicing (Supplementary Table 2). In agreement with the absence of candidate splicing variants, *LRRFIP2* or *MLH1* aberrant transcripts were not identified by RNA-seq (data not shown).

Insertion or rearrangements of *Alu* sequences were not detected within the DMR nor *MLH1* gene in any of the four CME carriers. Beyond these specified regions, we identified 11 heterozygous *Alu* insertions in CME carriers (Supplementary Table 3). Additionally, we detected 9 candidate structural variants (SVs), none of them within the DMR or *MLH1* gene body in the two relatives and in three CME carriers (Supplementary Table 4). Therefore, in agreement with previous reports on primary CMEs, our results suggest that neither SNVs, mobile elements nor SVs were implicated in the mechanisms underlying CME in the four patients included in this study.

### The epimutant allele features an inactive chromatin conformation at the *MLH1* promoter

**Fig. 1:**
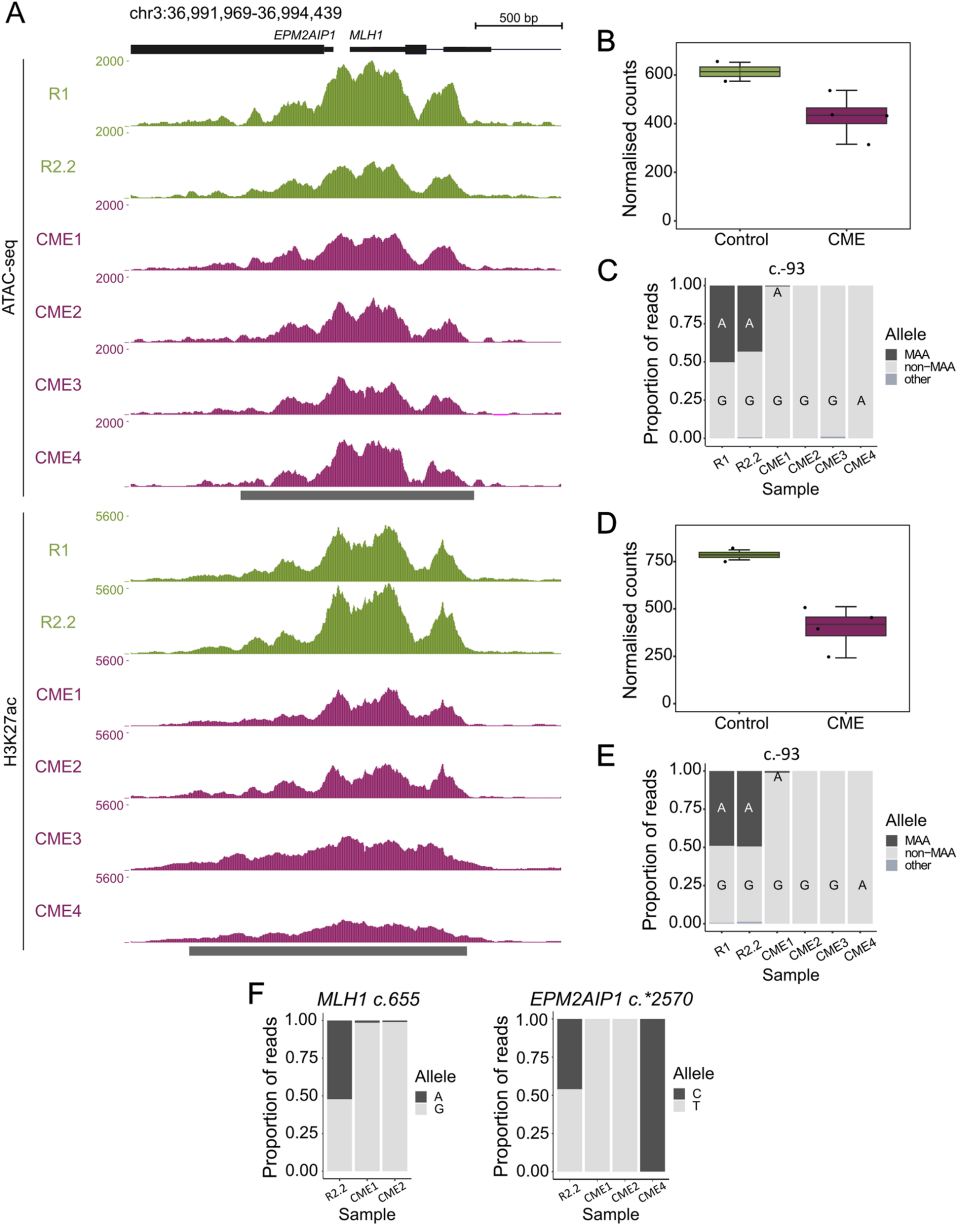
Characterisation of *MLH1* promoter and functional validation of the epimutations. **A** UCSC screenshot at chr3:36,991,969–36,994,439 showing the ATAC and H3K27ac enrichment signal at the *MLH1* promoter in each sample. Green and purple tracks represent control and epimutant (CME) cells, respectively. Below the peak profiles, the consensus peak region is indicated in grey. Normalised counts at the *MLH1* promoter for ATAC (**B**) and H3K27ac (**D**) peaks. Wilcoxon p value = 0.13. Proportion of ATAC (**C**) and H3K27ac (**E**) reads harbouring each allele at *MLH1* c.-93 (chr3:36,993,455). **F** Allelic expression of two common *MLH1* (left) and *EPM2AIP1* (right) SNPs in the heterozygous samples

In the absence of genetic alterations clearly linked to the CMEs, we next sought to explore whether the epimutant allele features altered regulatory functions related to the allele-specific *MLH1* loss of function. Thus, we first assayed chromatin accessibility by ATAC-seq in LCLs from the four CME carriers and the non-carrier relatives R1 and R2.2 (Fig. 1A). Compared to control cells, we observed that epimutant cells presented a reduced number of ATAC-seq normalised read counts mapping to the *MLH1* promoter suggesting decreased chromatin accessibility (Fig. 1B). We took advantage of a heterozygous reporter genetic variant (c.-93G > A) located within the *MLH1* promoter to determine whether the reduction in accessibility was predominantly associated with one of the two alleles. While in control cells ATAC-seq reads were mapped in similar proportions to the two alleles, in cells from CME carriers 98–100% of the chromatin accessibility signal came from the non-methylation-associated allele (non-MAA) (Fig. 1C). These data imply that in the epimutant cells the MAA promoter is in a closed conformation structure. Consistently, in CME3, the allele-specific accessibility was confirmed with an additional genetic variant (c.-234_-236del) in *trans* with the MAA (Supplementary Fig. 2A).

Next, we used CUT&Tag to map the deposition of H3K27ac, a histone modification typically associated with transcriptionally active chromatin. Consistent with ATAC-seq, we observed a reduction in the H3K27ac reads mapping to the *MLH1* promoter in epimutant cells as compared to controls (Fig. 1D). Again, after splitting the reads based on the heterozygous reporter genetic variant at c.-93, we observed discordant proportions of allele-specific H3K27ac enrichments in the cells of controls versus CME carriers and on the non-MAAs (Fig. 1E, Supplementary Fig. 2B). Overall, these data indicate that the MAA *MLH1* promoter lacks both accessibility and active chromatin marks exclusively in epimutant cells. Importantly, in the cells of the non-methylated controls, the same allele exhibited an active chromatin configuration.

We applied RNA-seq to gauge the impact of DNA methylation and inactive chromatin conformation at the *MLH1* promoter on gene expression activity in LCLs. We assessed the allele-specific expression of two common *MLH1* and *EPM2AIP1* SNPs, c.655G > A (rs1799977) and c.*2570 T > G (rs9311149), respectively. Most samples were heterozygous for at least one of these SNPs; however, CME3 and R1 were homozygous for both SNPs hence excluded from this analysis. As expected, while the control cells (R2.2) expressed both alleles at similar levels, the epimutant cells exhibited monoallelic expression (Fig. 1F). Our findings were consistent with the monoallelic expression previously observed in CME1 and CME2 primary lymphocytes [10].

Differences in chromatin accessibility and activity between control and CME cells were also analysed at the genome-wide level. Our principal component analyses showed an overall high similarity between the chromatin landscapes of the different cell lines (Supplementary Fig. 3A, B), suggesting that cancer predisposition in CME is linked to focal events rather than genome-wide chromatin remodelling. Of note, significant differences were observed in chromatin accessibility for three regions (Supplementary Fig. 3C, Supplementary Table 5) and in H3K27ac occupancy for four regions (Supplementary Fig. 3D, Supplementary Table 6), between the CMEs and controls, although none of the associated genes was found to be differentially expressed (Supplementary Table 7). We found only 52 differentially expressed genes in epimutant cells compared to control cells (Supplementary Fig. 3F, Supplementary Table 7). Among them, we identified 3 TFs that were upregulated (*BHLHA15, NR2F2, PMEPA1*) and 3 downregulated (*EFNA5, RSC1A1, SGMS2*) in epimutant cells compared to the controls. We scanned the *MLH1* promoter and found that 2 of the upregulated TFs had binding sites within the *MLH1* promoter, namely NR2F2 and BHLHA15, both with the potential to act as repressor TFs [69, 70].

### Wild type and epimutant alleles showed allele-specific promoter interactions

**Fig. 2:**
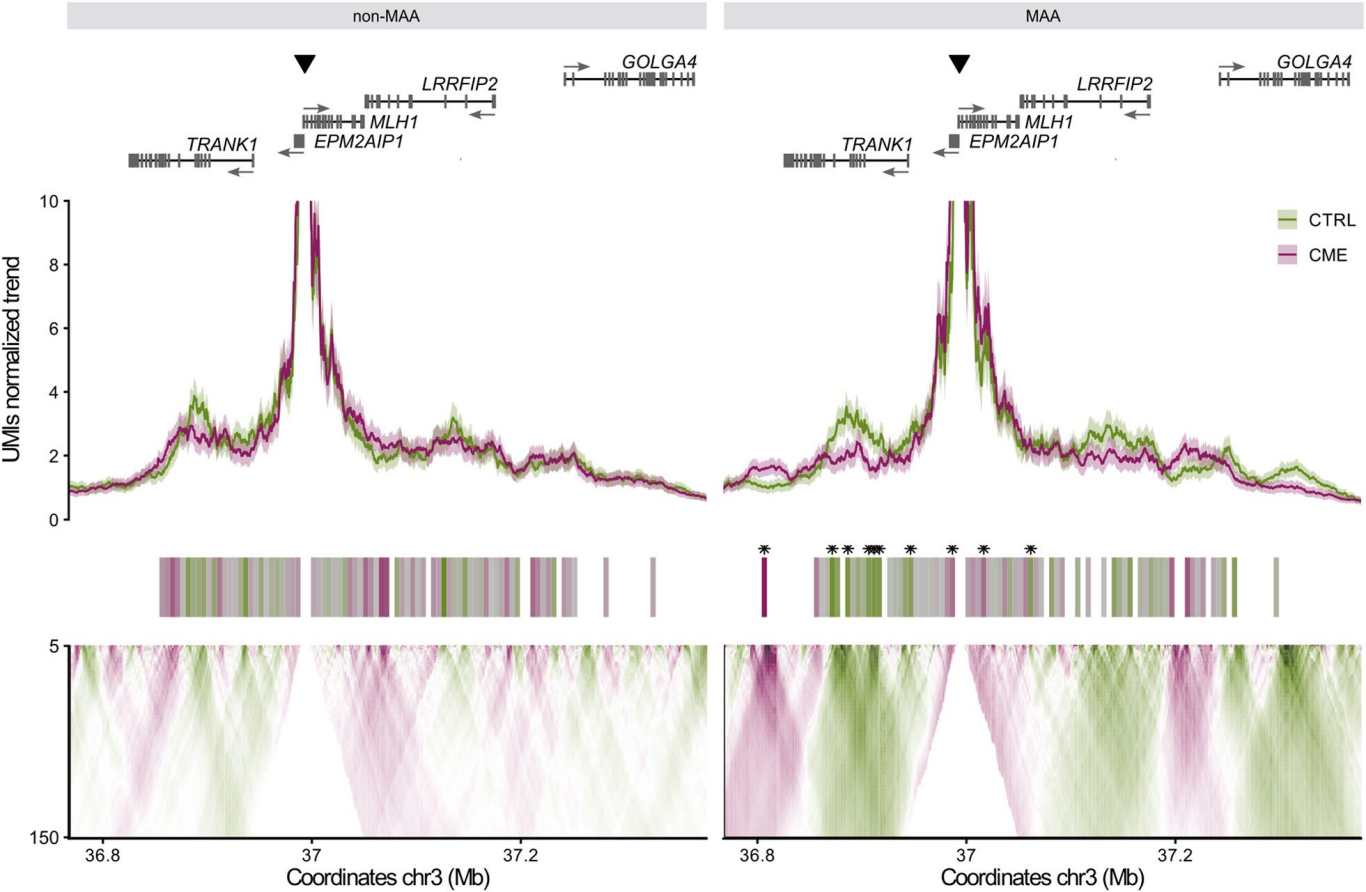
Contacts of the *MLH1* promoter. Profiles of *MLH1* promoter contacts in control (CTRL, green) and epimutant (CME, purple) cell lines for the non-MAA and the MAA. The viewpoint is indicated by the black triangle. Protein coding gene annotation and transcriptional direction are shown at the top. Below, the UMI-4C contacts normalised trends and its corresponding windows, with fill representing the log_10_ odds ratio of the contact differences in epimutant versus control cells. Asterisks on top of the windows indicate significant differences in the 3D contacts (FDR adjusted *p*-value < 0.05). The domainogram at the bottom shows the mean contact intensity log_2_ fold changes with increasing resolution between the two groups. Colour scale is the same in all graphs: green and purple indicate lost and gained contacts, respectively, in epimutant cells compared to controls

We next explored whether the differences in chromatin accessibility and activity between alleles were linked to allele-specific differences in the 3D chromatin interactions. To address this question, we developed an allele-specific UMI-4C assay taking advantage of the c.-93 reporter variant. Importantly, we chose to use the UMI-4C technique for this analysis to achieve a quantitative comparative assessment of the chromatin contacts, which is not guaranteed with other chromatin capture techniques such as regular 4C that are prone to PCR amplification biases. By careful comparison and strict statistical analyses of allele-specific UMI-4C data, we did not observe significant differences in the interactions profile of the two (unmethylated) c.-93 alleles in control cells (Supplementary Fig. 4). Strikingly, we observed a different 3D chromatin contact profile of the MAA when comparing control versus epimutant cells. In contrast, the non-MAA showed non-statistically significant differences when comparing epimutant and control cells (Fig. 2). The eight regions displaying statistically significant changes in MAA included gained and lost interactions (Fig. 2, right). The differential contacts and the nearby regions were marked by the presence of ATAC and H3K27ac peaks (Supplementary Fig. 5A, B). In fact, a permutation test showed that the differential contacts overlapped with accessible regions more than expected by chance (Supplementary Fig. 5A), indicating that the differential contacts likely harbour distal regulatory elements implicated in the regulation of *MLH1* gene expression.

### Genetic characterisation of *MLH1* promoter contacts identified variants predicted to alter transcription factor binding sites

Regulatory elements harbour binding sites for TFs. Genetic variants mapping to these regions can thus interfere with TF binding, resulting in modulation of gene expression [71], and can influence the local epigenetic state. We hypothesised that some regions might be more prone to accumulate variants than others. To explore whether genetic variants could potentially influence *MLH1* silencing, we searched for rare variants mapping to regulatory regions identified by UMI-4C to be in physical contact with the *MLH1* promoter. By using a sliding window approach, we uncovered an upstream differential contact exhibiting a high frequency of rare variant accumulation in CME carriers (Supplementary Fig. 5B). Specifically, a total of 99 distinct variants were found in the CME carriers, of which 34 were in phase with the MAA (Supplementary Fig. 5C). Interestingly, 31 of these 34 in-phase variants were predicted to alter transcription factor binding sites (Supplementary Fig. 5C, Supplementary Table 8). Eleven of these variants (11/31, 35.5%) came from a specific sample (CME3), which overall accounts for a higher number of rare variants in the promoter-contacting regions compared to the other carriers (Supplementary Fig. 5D). We did not find variants with a strong effect on insulator protein binding sites in any of the CME carriers. In contrast, we identified 8 rare variants whose alternative genotype was predicted to facilitate the binding of repressor TFs by enhancing similarity to their binding motifs, and 14 rare variants predicted to decrease binding affinity of an activator TF, although none of them were located within the promoter differential contacts (Supplementary Fig. 5C, Supplementary Table 8).

None of the SVs and *Alu* insertions found in our previous analyses were located within the *MLH1* promoter-contacting region.

Overall, in *cis* genetic alterations potentially affecting TF binding sites at *MLH1* promoter contacts that may have a role in predisposition to *MLH1* silencing were identified in the four CME carriers included in this study.

## Discussion

**Fig. 3:**
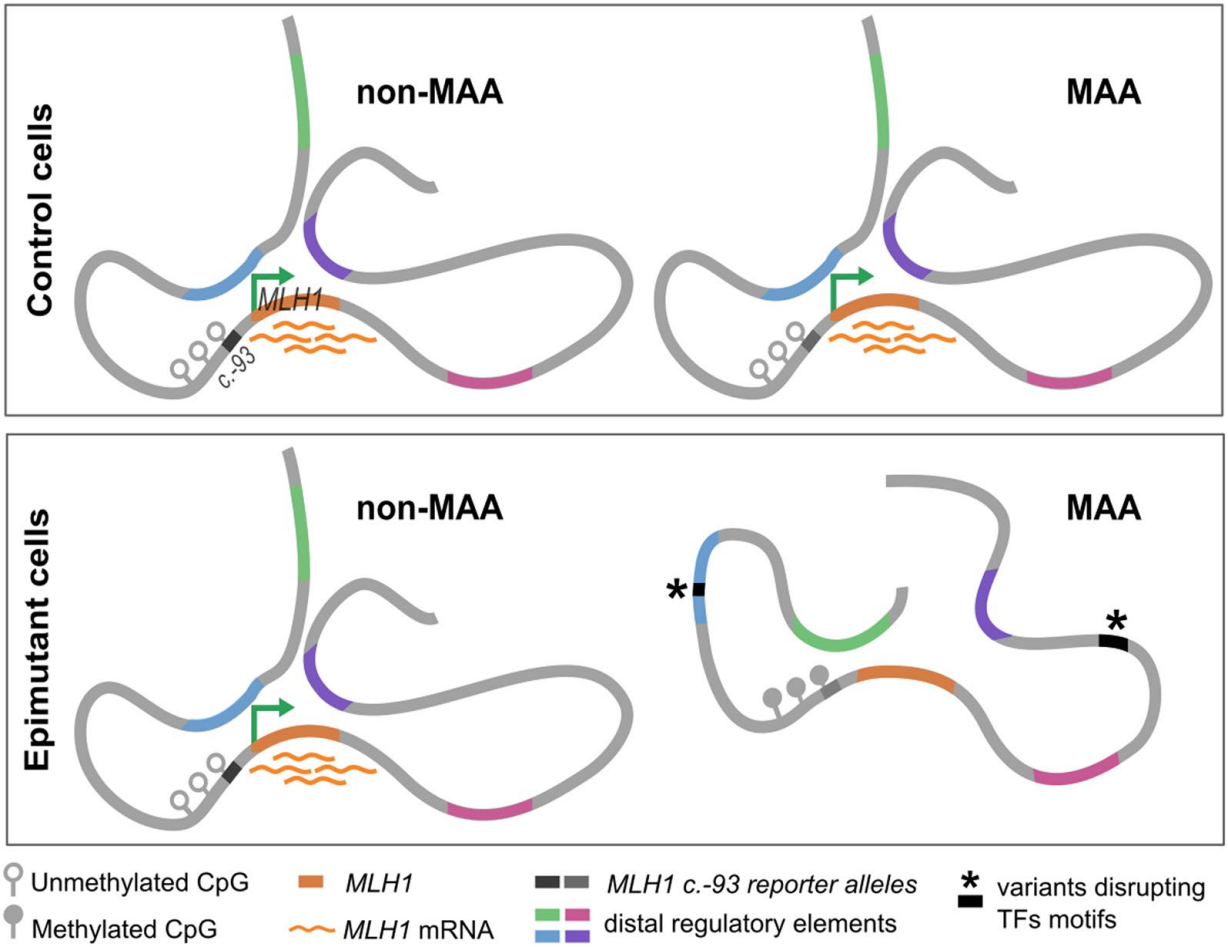
Schematic representation of the changes in 3D contacts and expression associated with *MLH1* promoter methylation. In control cells (derived from CME’s relatives), *MLH1* promoter is unmethylated and both alleles show the same pattern of interactions regardless of the c.-93 genotype, with concomitant biallelic expression of *MLH1*. In cells carrying the epimutation (epimutant cells) the MAA allele is methylated independently of the c.-93 genotype. *MLH1* methylation and loss of transcription are coupled to changes in the 3D promoter interactions. Regulatory elements in physical contact with the *MLH1* promoter can harbour genetic variants in *cis* with the MAA that may alter binding of TFs and could predispose to *MLH1* epimutation

Here, we have characterised the chromatin landscape associated with the CME in four selected patients who carried a “primary” CME. For the first time, we have shown that, compared to the *MLH1* promoter unmethylated allele, the constitutional MAA is less accessible and active. These observations are consistent with monoallelic loss of expression of *MLH1* and *EPM2AIP1*, both genes regulated by the same promoter. Moreover, we have demonstrated that the promoter MAA exhibits differential 3D contacts compared to the non-MAA, including loss and gained interactions with distal regions. Finally, rare in *cis* genetic variants affecting the binding of repressors and activators were identified within the promoter-contacting region in the four CME carriers, which may have a role on predisposing the allele to *MLH1* methylation (Fig. 3).

Typically, CME carriers have been screened for in *cis* variants within the *MLH1* promoter region. The four CME carriers studied here were previously screened for the presence of variants within the DMR [10, 72]. In the present work, we excluded the presence of shared rare point genetic variants in the entire *MLH1* and *LRRFIP2* genes. Instead, a limited number of rare intronic variants not predicted to impact splicing were identified in each case. Consistent with this, RNA-seq analysis also did not identify any aberrant transcripts. Similarly, insertions or rearrangements of *Alu* sequences or SVs, previously found in index cases with a secondary CME [4, 14, 73, 74], were also ruled out. Of note, structural rearrangements can modify chromatin topology and are associated with aberrant methylation of CpG islands [75–78]. Neither mobile element insertions or rearrangements nor SVs were detected within the DMR, across the *MLH1* gene, or the distal regions in physical contact with the *MLH1* promoter in the four CME carriers. Overall, the lack of any findings of singular genetic abnormalities in *cis* the epimutation in these four CME carriers is in agreement with the classification of “primary” CME.

We have profiled changes in chromatin landscape at the genome-wide level and identified only a few regions exhibiting differential accessibility or H3K27ac enrichment in CMEs as compared to control cells. This observation is consistent with the minimal transcriptional differences found in the cells from the CME and control groups and the focal nature of the CME. Of note, the *MLH1* promoter contains binding motifs for two TFs, NR2F2 and BHLHA15, that we identified as differentially expressed between the CME and control cells. NR2F2 can either act as a transcriptional activator or repressor in a gene-specific manner to regulate developmental processes, such as angiogenesis, adipogenesis and neural differentiation [69]. On the other hand, BHLHA15 encodes for a transcription factor whose mouse orthologue Mist1 is expressed in post-implantation E10.5 embryos [70]. Despite the potential for both TFs to act as transcriptional repressors, there is no reported evidence of association with DNA methylation.

The overall small number of transcriptional and chromatin changes was expected, as the LCLs used in this study were derived from healthy lymphocytes obtained from CME carriers whose methylation pattern only differs from their control counterpart at the *MLH1* locus [10]. The small differences observed between our CME and control cell lines may be caused by cell diversity and clonal evolution of LCLs through culturing, as previously reported [79, 80]. Nevertheless, differential analysis of chromatin accessibility, H3K27ac enrichment or gene expression performed on distinct cell groups may fail to capture allele-specific features. Allele-specific differences were evident when we could take advantage of informative heterozygous variants within the *MLH1* promoter (c.-93G > A and c.-234_-236del) that uncovered differential chromatin accessibility in the ATAC and H3K27ac reads, and monoallelic expression of exonic *MLH1* (c.655) and *EPM2AIP1* (c.*2570) SNPs in the RNA-seq data.

Using our own UMI-4C approach, we could perform a quantitative allele-specific analysis and identify changes in the 3D contacts between the MAA and non-MAA in CME LCLs. These changes are mainly linked to the promoter methylation state, as both alleles in the control cell lines exhibited the same pattern of 3D contacts. A previous study suggested that the c.-93A variant may induce increased contacts between the *MLH1* promoter and the *DCLK3* gene resulting in its enhanced expression in CRC cell lines [81]. Based on our findings in LCLs, the observed changes in 3D contacts cannot be attributed to the c.-93A variant itself, suggesting the *MLH1* promoter-*DCLK3* contact may be restricted to CRC cell lines due to the binding of colon-specific factors not present in patient-derived LCLs.

The differential contacts identified when the two alleles (MAA and non-MAA) were compared likely contain regulatory elements that modulate *MLH1* expression. Of note, two of the eight differential contacts identified in our study include previously reported putative regulatory regions that correlate with *MLH1* expression across distinct cell types: a positive correlation in one and a negative correlation in the other [82]. Interestingly, the region that negatively correlates with *MLH1* expression was found to have gained contacts with the MAA in epimutant cells compared to controls. Conversely, the region that positively correlated with *MLH1* expression showed a loss of physical contacts on the MAA compared to the unmethylated allele.

Variants in distal regulatory elements influence chromatin contacts and gene expression [71, 83], and correlations between genetic variants and DNA methylation states exist, with 10–45% of the methylome being influenced by nearby genetic variants [84]. These variants tend to accumulate in non-genic regions and enhancers, while being depleted in CpG islands, 5’UTRs and regions upstream of transcription start site. Considering this, we hypothesise that variants located within the differential contacts might predispose to *MLH1* methylation (Fig. 3). Although we did not identify any in *cis* genetic variants predicted to alter binding of activators or to enhance binding of repressor factors inside the differential contacts, we did detect in *cis* variants affecting motifs in all CME carriers across the promoter-contacting region. It is important to note that we could not determine the phase for all the detected variants within the promoter-contacting region, particularly in CME2, for which the phase block including the c.-93G > A reporter SNP did not cover the entire promoter-contacting region. Additionally, we only used four primary CMEs and two control relatives to determine the differential contacts, which limits the statistical power of the analysis. Our study represents the first comprehensive genetic and epigenetic (ATAC-seq, CUT&Tag, UMI-4C) characterisation of primary CME carriers using peripheral blood lymphocytes and derived LCLs. Furthermore, we have performed allele-specific analyses to investigate the alterations directly coupled with CME. The fact that the *MLH1* promoter is the only differentially methylated region in “primary” CME cases indicates a focal defect, for which one hypothesis is that the existence of *cis* elements makes it prone to become methylated under certain environmental conditions.

The main limitation of our study lies in the use of LCLs. Primary epimutations likely arise at the preimplantation phase during embryo development or at the establishment of the DNA methylation pattern phase during germ cell maturation [8], stages in which the *cis*-regulatory landscape at the *MLH1* locus may differ from that observed in LCLs. Future studies should consider using induced pluripotent stem cells that have undergone generalised DNA demethylation to capture the contribution of regulatory genetic variants to the establishment of a transcriptional silencing conformation of the *MLH1* promoter. Regarding technical limitations, we used short-read sequencing data to infer SVs and transposable element insertions. However, long-read sequencing methods have shown greater potential to identify these sorts of variants [89, 90]. Applying long-read sequencing could result in the identification of additional genetic alterations affecting in-*cis MLH1 r*egulatory regions.

In conclusion, by using a very comprehensive (epi)genetic approach, we have identified putative regulatory regions that might influence the epigenetic state of the *MLH1* promoter. Also, we have explored genetic variants within the promoter-contacting regions which might confer susceptibility to the epimutation. As a result, our study provides valuable *cis*-regulatory maps that will facilitate the discovery of genetic variants implicated in *MLH1* silencing. Further mechanistic studies, such as in pluripotent stem cells, are needed to elucidate the functional impact of the identified *cis*-regulatory regions in the origin and/or maintenance of CME.

## Supplementary Information

Additional file 1.

Additional file 2.

Additional file 3.

## Data Availability

ATAC-seq, H3K27ac CUT&Tag, UMI-4C, RNA-seq and WGS data generated in this publication are deposited at the European Genome-phenome Archive at the European Bioinformatics Institute with the IDs EGAD50000000714, EGAD50000000711, EGAD50000000713, EGAD50000000712, and EGAD50000000710, respectively.
