# Supplementary material for: Altered chromatin landscape and 3D interactions associated with primary constitutional MLH1 epimutations
Source: Clin Epigenetics. 2024 Dec 31;16:193. doi: 10.1186/s13148-024-01770-3 (PMC11686911; doi:10.1186/s13148-024-01770-3)

SUPPLEMENTARY FIGURE LEGENDS

Supplementary Figure 1. Pedigrees of the CME cases. CMEs are indicated with an arrow. CMEs and relatives included in the study are labelled with the corresponding ID in bold letters, their current age is indicated below. Co-segregation of epimutation was studied in Families 1, 2 and 4, where lack of co-segregation was shown. Haplotypes are schematised by sticks: Red=methylation-associated allele (MAA), Black=haplotype also carried by the proband but not associated with the epimutation, Blue=un-informative haplotypes. The presence of methylation (M) or its absence (UM) is indicated on the red MAA haplotypes for those individuals tested. Generations are indicated on the left margin in Roman numerals and analysed relatives are identified by numbers. Circles, females; squares, males; filled, cancer affected. OC, ovarian cancer; BrC, brain cancer; CRC, colorectal cancer; CC, colon cancer; EC, endometrial cancer; KC, kidney cancer. Pedigrees were adapted from Dámaso et al. 2018.

Supplementary Figure 2. Characterisation of *MLH1* promoter. Proportion of ATAC (A) and H3K27ac (B) reads harbouring each allele at the *MLH1* c.-234_-236 deletion (read count at chr3:36993314).

Supplementary Figure 3. ATAC, H3K27ac CUT&Tag, and RNA-seq differential analysis. A-B) PCA based on DESeq2 VST normalised counts from ATAC-seq (A) and H3K27ac CUT&Tag (B) data. Circles and triangles depict control and epimutant (CME) cells, respectively. Individuals from the same family are indicated with the same colour. C-D) Volcano plots showing differentially accessible regions (C) and differentially H3K27ac enriched regions between control and epimutant (CME) cell lines. Changes with adjusted p-value (padj) <0.1 are coloured in salmon and green when showing lost or gained chromatin accessibility or H3K27ac content, respectively. Regions with padj >0.1 are considered as stable (blue). E) PCA of RNA-seq data. Circles depict controls and triangles epimutant cells. Individuals from the same family are indicated with the same colour. F) Volcano plot showing differentially expressed genes between control and epimutant (CME) cell lines. Genes with lost or gained expression are marked in salmon and green, respectively. Genes with stable expression across conditions are depicted in blue.

Supplementary Figure 4. Differential promoter contacts in control cells. Profiles of UMI-4C *MLH1* promoter contacts for each allele captured by the c.-93 reporter variant genotype (G, A) in control cells. The A allele is shown in green and the G allele in purple. Genes inside the contact region are indicated at the top. In the middle, UMI-4C contacts normalised trends and window representing the $\log_{10}$ odds ratio of the contact differences between the two alleles in control cells. Gained and lost contacts of the A allele are indicated in green and purple respectively. No statistically significant differences were detected between the G and A allele (FDR adjusted p-value > 0.05).

Supplementary Figure 5. Distribution of genomic features. A) Boxplots represent the random distribution of overlaps between ATAC or H3K27ac peaks and UMI-4C differential regions obtained by permutation tests (n=500) and diamonds depict the observed number of overlaps. Green diamond indicates statistically significant differences (p-value < 0.05) between random and observed events. B) Density of variants around *MLH1*. The density of variants was computed applying a sliding window approach using overlapping windows of 20 Kb and steps of 5 Kb. Each point represents the number of variants per patient in each window. Below, protein coding genes, the differential UMI-4C regions (gained and lost contacts in purple and green

respectively), and ATAC and H3K27ac peaks overlapping the analysed region. C) Summary of rare variants found in each CME carrier. D) Number of rare variants found in each sample.

Supplementary Figure 1

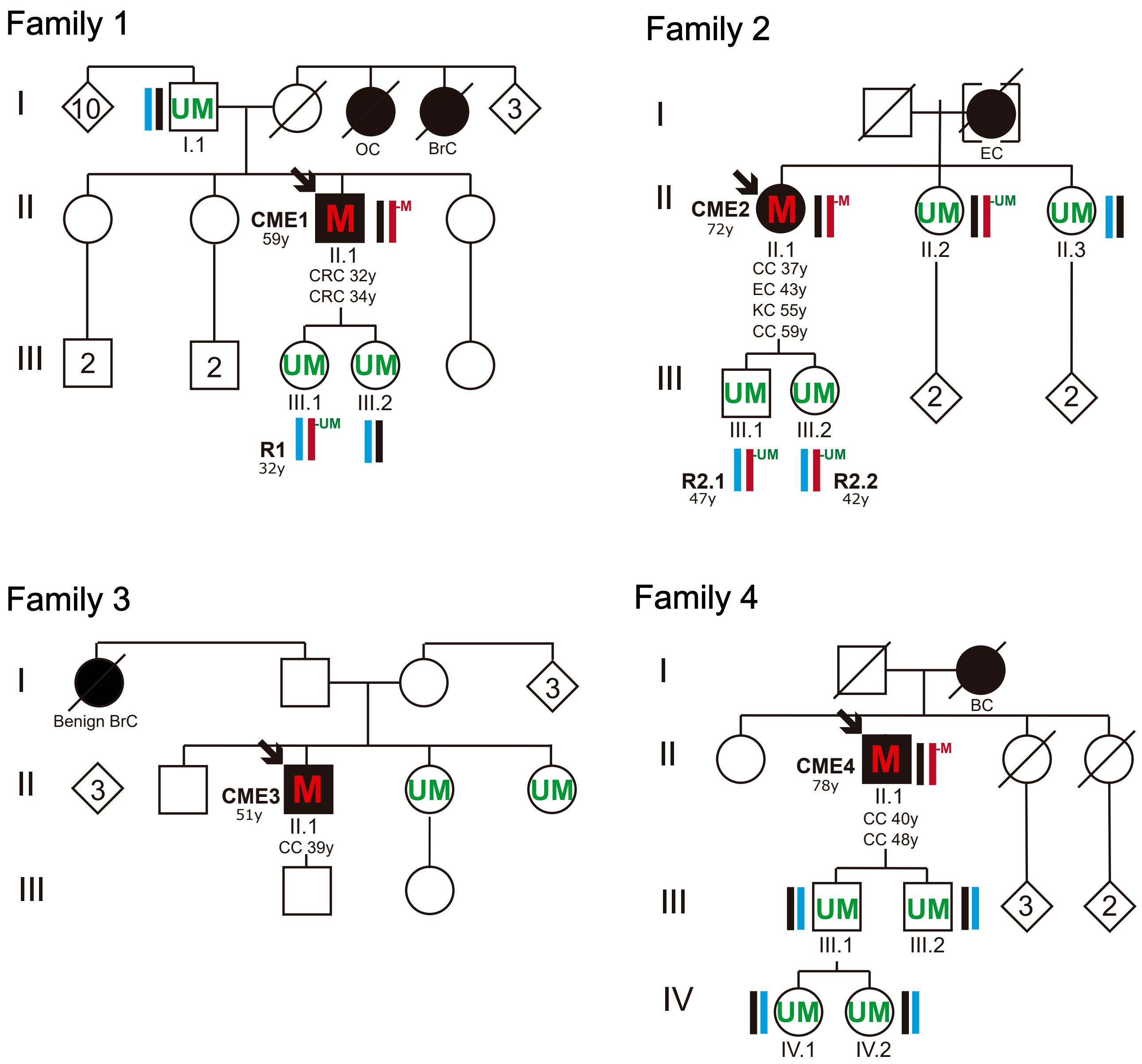

Supplementary Figure 2

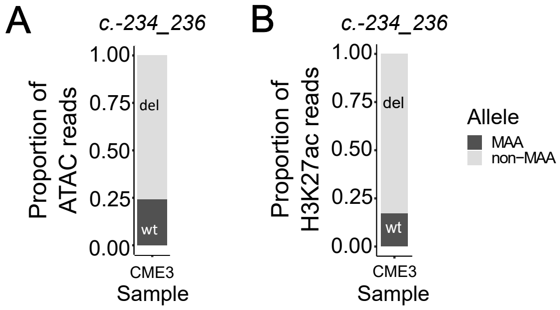

Supplementary Figure 3

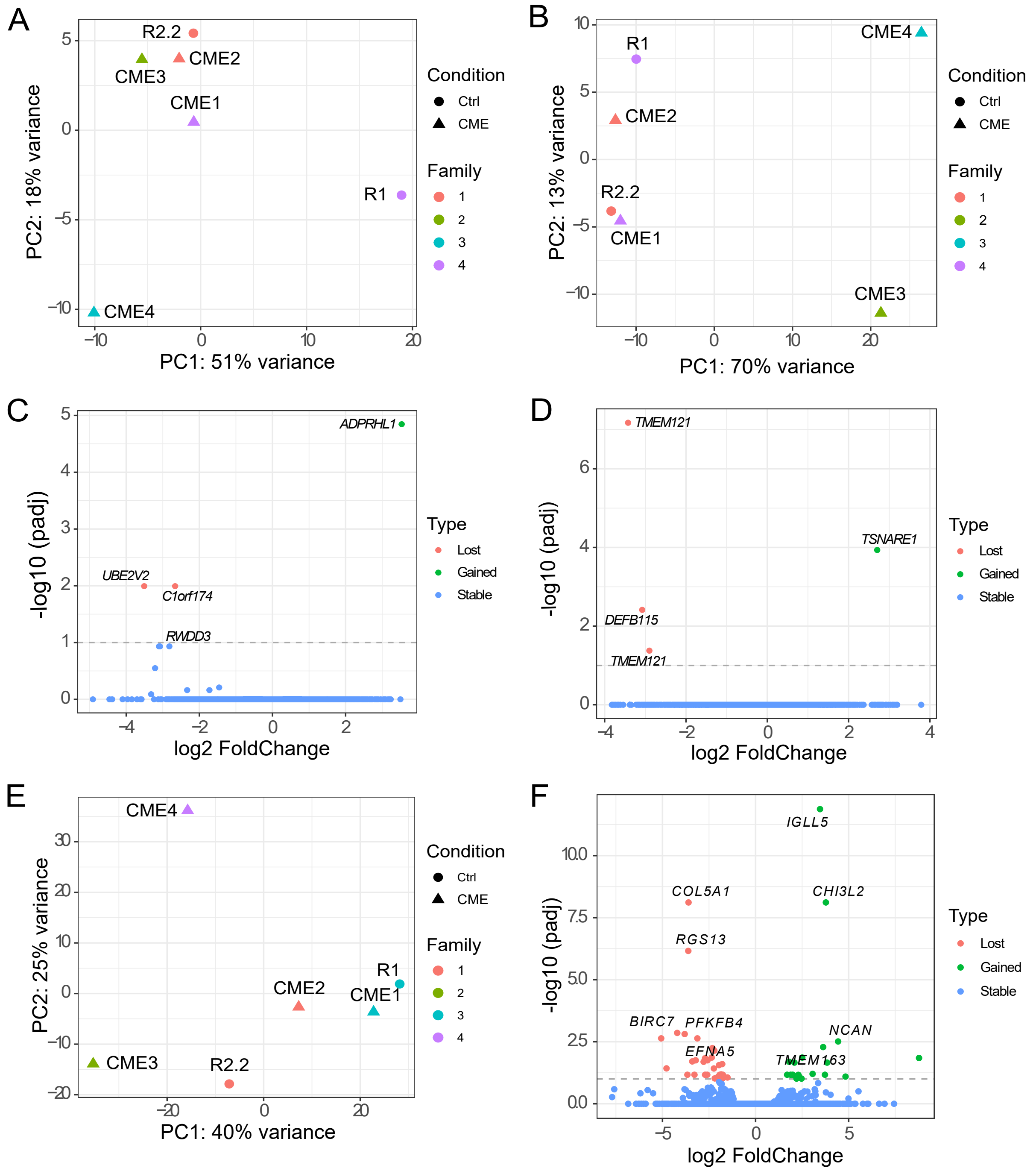

Supplementary Figure 4

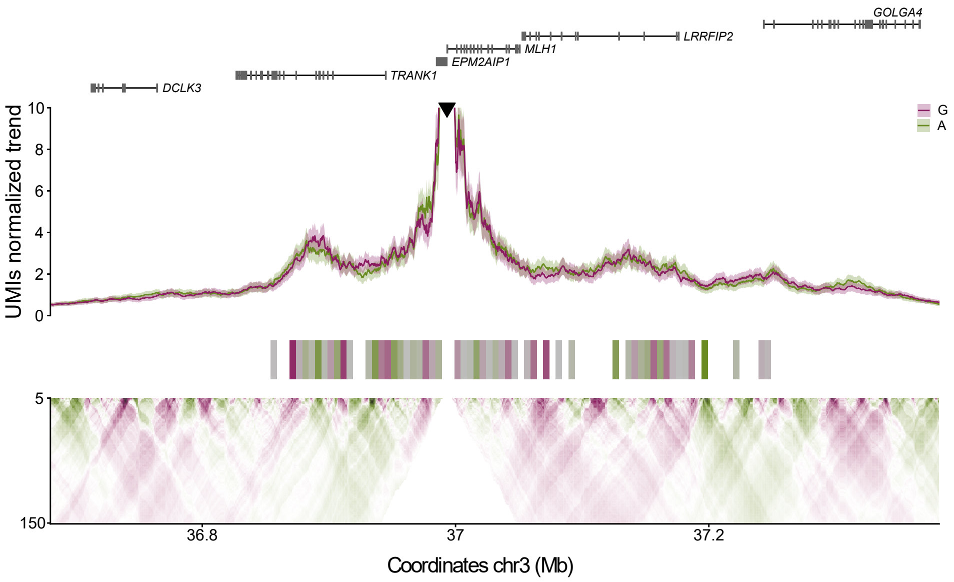

Supplementary Figure 5

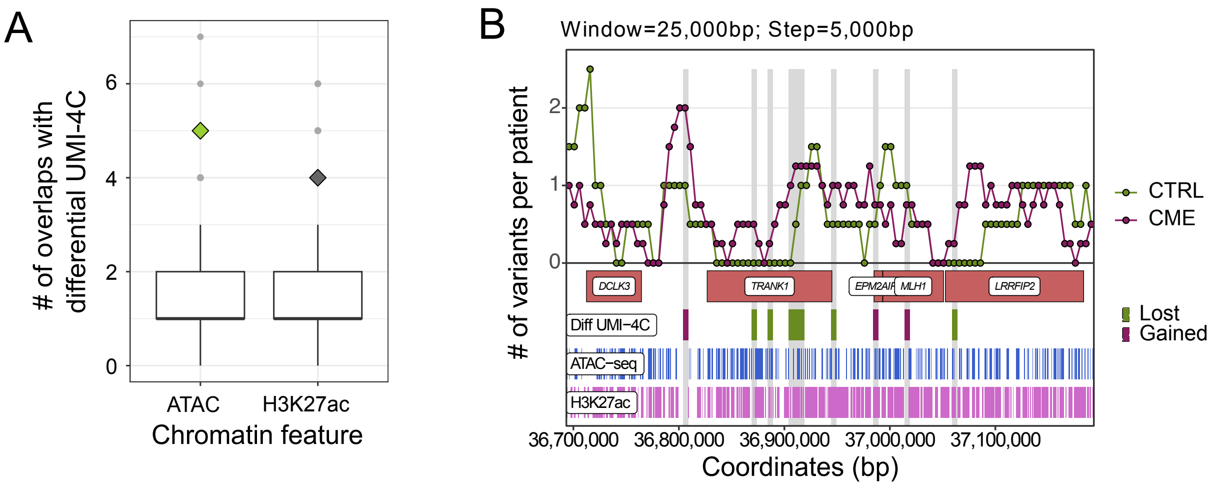

C

	CME1	CME2	CME3	CME4
Variants inside the promoter contacting region	27	16	43	13
Variants in phase with the MAA	8	5	13	8
Variants in phase with the MAA with effect on TF binding sites	8	4	11	8
Variants in phase resulting in gain of repressor binding	2	0	5	1
Variants in phase resulting in loss of activator binding	2	2	6	4

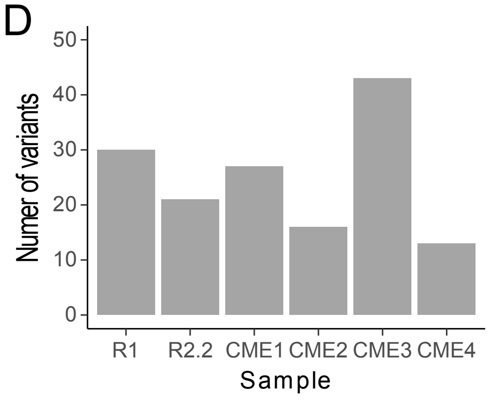

Supplement: Supplementary file 1 — Additional file 1. [file 13148_2024_1770_MOESM1_ESM.pdf]
