# Supplementary material for: Altered chromatin landscape and 3D interactions associated with primary constitutional MLH1 epimutations
Source: Clin Epigenetics. 2024 Dec 31;16:193. doi: 10.1186/s13148-024-01770-3 (PMC11686911; doi:10.1186/s13148-024-01770-3)
Supplement: Supplementary file 2 — Additional file 2. [file 13148_2024_1770_MOESM2_ESM.pdf]

SUPPLEMENTARY TABLE LEGENDS

Supplementary Table 1. Allelic frequency and splicing prediction of heterozygous *MLH1* intronic variants. Population frequency (AF, gnomAD v3.0) of the heterozygous *MLH1* intronic variants reported in Table 1. Effect on splicing was calculated using SpliceAI. The deltaScore for each possible acceptor or donor event in the MANE transcripts is shown. None of the variants was predicted to alter splicing. Rare variants in *MLH1* shared between CMEs and their relatives are indicated in bold.

Supplementary Table 2. Rare heterozygous variants identified inside *LRRFIP2*. Rare variant (AF <1%) identified in CME carriers and relatives. Genomic coordinate (hg38 position), name according to HGVS guidelines and using MANE transcript NM_006309, intronic region, SNP ID, and allele frequency (AF, gnomAD v3.0) are indicated. Rare variants shared among the CME and their relatives are indicated in bold. Effect on splicing was calculated using SpliceAI. The deltaScore for each possible acceptor or donor event in the MANE transcripts is shown. None of the variants was predicted to alter splicing. UP=unphased; Un=unknown, variants in different phase blocks.

Supplementary Table 3. Heterozygous *Alu* insertions identified in epimutation carriers. The table contains information about chromosomal position (POS), target site (TSD), and length of insertion, Alu subfamily, relative start and end of the inserted region, and insertion polarity. Genes affected by the insertion are indicated. Insertions found in more than one sample are indicated in bold. Null: not able to determine affected genes.

Supplementary Table 4. SVs found in the epimutation carriers and their relatives. For each SV the start and end coordinates of the SV and width are indicated. SV type and number of callers supporting each SV are indicated (DEL: deletion, DUP: duplication, INV: inversion, INS: insertion, BND: breakend). The number of counts supporting the SV in each sample and in the population (Pop) is detailed. Last columns contain the Ensembl IDs and gene symbols from genes overlapping each SV.

Supplementary Table 5. Results from the ATAC-seq differential analysis. For each peak, the coordinates of the peak are indicated. Results of the DESeq2 analysis between epimutant and control cells, including the $\log_2$ fold change, p-value, and adjusted p-value (padj). The nearest gene to each peak and the distance to the peak are included. Peaks are classified according to their proximity to the nearest gene (annotation) as “distal” (located more than 2 Kb away from the nearest gene) or as “promoter” (within 2 Kb of the gene). Considering the changes in accessibility, those peaks showing statistically significant changes (padj <0.1) were classified as “gained” or “lost”, the rest were considered as “stable”.

Supplementary Table 6. Results from H3K27ac CUT&Tag differential analysis. For each peak, the coordinates of the peak are indicated. Results of the DESeq2 analysis between epimutant and control cells, including the $\log_2$ fold change, p-value, and adjusted p-value (padj). The nearest gene to each peak and the distance to the peak are included. Peaks are classified according to their proximity to the nearest gene (annotation) as “distal” (located more than 2 Kb away from the nearest gene) or as “promoter” (within 2 Kb of the gene). Considering the changes in accessibility, those peaks showing statistically significant changes (padj <0.1) were classified as “gained” or “lost”, the rest were considered as “stable”.

Supplementary Table 7. Results from RNA-seq differential analysis. List of genes differentially expressed in epimutant cells compared to controls. For each gene, Ensembl ID and output from

DESeq2 analysis including log_2 fold change, p-value, and adjusted p-value (padj) are shown. Genes were classified as “gained” or “lost” according to their fold change when padj <0.1. Genes with padj >0.1 were annotated as “stable”.

Supplementary Table 8. List of variants fount in CME carriers with a predicted strong effect on transcription factor binding sites. For each variant, it is detailed the sample where it was found and its genomic coordinates, reference (Ref) and alternative (Alt) alleles as well as motifbreakR output. The motifbreakR output includes the motif strand, factor recognising the motif (geneSymbol), motif information from the source (dataSource, providerName, providerID, seqMatch), normalised allele scores (pctRef, pctAlt), allele scores (scoreRef, scoreAlt), difference between scores (alleleDiff), and effect of the alleles (alleleEffectSize). Genotype and phase block of the variants are indicated; those variants in phase with the methylated allele are coloured in blue. The last two columns indicate if the TFs are classified as repressors/activators (TRUE) or not (FALSE).
